# Failure of Fillings

**Published:** 1888-02

**Authors:** G. S. Staples

**Affiliations:** Sherman, Texas.


ARTICLE III.

### FAILURE OF FILLINGS.

BY G. S. STAPLES, D. D. S., SHERMAN, TEXAS.

[Read before the Southern Dental Association, Old Point Comfort, Va., September 1, 1887.

Finding my name on the list of names composing your committee on operative dentistry, my first impulse was to make no attempt myself, but leave it to "the other fellow" to prepare a paper. But, as I know, we are too much in the habit of waiting for "the other fellow" in such matters. I, after reflection and through solicitation of our worthy chairman, decided to prepare a paper, whether I said anything or not.

As I made a few remarks during the discussion of the failure of fillings, at Nashville, last year, which seemed to have stirred up a "little breeze" among some of the members, and as I am satisfied my meaning was misconstrued at the time, I have selected the subject of "Failure of Fillings" for my paper.

Now, gentlemen, I do not propose to make any apologies for what I may say in this, for I have to be allowed to talk plainly, or not talk at all. I only propose to give you my own humble opinion for what it is worth; just that and nothing more. So, should I happen to hit any one on a tender spot, just clap your hands on the spot until it quits hurting, and take it in as good part as it was meant.

If there is any one subject pertaining to dentistry that I have given more thought than any other. it is the subject of failure of fillings. And I have arrived at the conclusion long since (and become more convinced of the fact every day), that more than ninety-five per cent. of failures is due to lack of thoroughness. So I propose to enumerate what I perceive to be some of the principal points of "lack of thoroughness." The first instance is the lack of thorough judgment on the part of tutors in selecting material for dentists. I believe the first, greatest and by far the most frequent cause is not, as our "Old" New Departure friends would say, due to electro-chemical incompatibility of the filling material to dentos; but rather due to electro-magnetic incompatibility between operator and patient, and positive incompatibility between the operator and his work. To repeat an oft-repeated saying (and one which none was ever truer) dentists are born, not made, and he who thinks he can take a young man, and because he has a very good education and an average amount of intelligence on general subjects, but no natural turn for dentistry, and make a first-class dentist of him, will find himself very much mistaken, and his pupil lacking in thoroughness all the way through.

Then comes a class of men who were born slipshod, raised slipshod and everything they do is slipshod, and, although they have the natural ability, the r entire lack of thoroughness makes their practice almost a failure Next comes a class of men who were born stingy, and who seem never to have cultivated any other crop through life, and so the natural result of their stinginess is lack of thoroughness in all their operations. They commence to prepare a cavity, and that they may use as little filling material as possible (especially if it be gold), they fail to thoroughly cleanse the cavity, for fear of getting it too large, and then they fail to consolidate the filling in order to economize again. And right here, gentlemen, I am sometimes afraid that this class is more numerous than most of us would like to believe.

Then comes the "all gold crank," who, because of his lack of thoroughness in manipulating other filling materials, decides that nothing is fit to fill teeth with except gold, and so uses gold in all cases.

How often do we see splendid gold fillings in mere shells of teeth, fit monuments to attest the superior skill of the operator; but alas for the preservation of those shells, it is but a few weeks or a few months at best, until the thin walls crumble away, leaving those fine fillings standing solitary and alone, like Sherman's chimneys in Georgia. Seeing such teeth filled with gold always reminds me of a little Frenchman who used to practice dentistry in our country when I was a small boy. In those days dentistry was not the scientific profession that it is to-day (as we all know). So the Frenchman always endeavored to impress it on you that he was something more than "von leettle denteest." So, in a crowd one day, while expatiating on his own accomplishments, he remarked: "Me no leettle denteest; me von operative surgeon; me excise ze enferior maxilary von, two, tree time. Beautiful operation! beautiful operation!"

"Well, Doctor," says a bystander, "how did you succeed, and how did your patients do?"

"Oh, pye gar, ze patient dey dies, but it vas von beautiful operation! von beautiful operation!"

Just so with the "all gold crank." He would waste valuable time and material, and sacrifice the tooth, just simply for the pleasure of seeing the result of "von beautiful operation."

Then come our very best operators, and they too make failures. Then they begin to look around for the causes of failure. One will fill a tooth with cohesive gold, and it fails. Another tells him if he had used non-cohesive it would have been better, and still another would advocate amalgam, and so on; the theory advanced being that the other materials being more plastic are consequently more easily forced into all parts of the cavity than cohesive gold, and so requiring less care to manipulate them. But having failed with them all (and it being human nature to look to everything but self for failures), he finally concludes it was the tooth at fault, and nothing would have saved it. But right there is where he makes the mistake in not going far enough with his investigations to discover the true cause of failure. I am sure that, had he gone a little further (in the great majority of cases), he would have discovered the true cause of failure to be lack of thoroughness in some part of the operation. Either the cavity was not thoroughly prepared, or in filling the plugger didn't reach every portion of the cavity, hence the natural result—failure. And right here, before closing this paper, I would call attention to one of the most fruitful causes of failure, "the retaining pit." Some of the worst failures, I believe, are caused by deep pits, severing the thin walls from all points of nourishment and thereby causing them to dry and crumble. I claim that no one can do strictly first-class work without an assistant, and with one to do the malleting, allowing me to use my left hand to hold the gold in place until I get it thoroughly anchored. I find no use for pits.

So what we want is thoroughness at every point. The thorough application of the business end of thorough dental instruments to every part of the cavity, with a thorough dentist at the other end, means success. Anything else will always result in failures. So, first be sure you understand your business, then be sure you have thorough instruments, then make thorough use of them.

When that is the case, then we will hear less complaint of failures of fillings, and less abuse of the materials used for filling teeth.—*Southern Dental Journal.*

